# War Emergency and Medical Education

**Published:** 1918-12

**Authors:** 


### WAR EMERGENCY AND MEDICAL EDUCATION

At the time the United States entered the world war until a few months ago, there were grounds for the belief that the war might extend over an indefinite number of years. That, indeed, was the only safe assumption the Government could take in its plans for carrying on the war. Following the extension of the selective service regulations to include practically all men of college age, units of the Students' Army Training Corps were established in the approved colleges of arts and sciences. Later the War Department decided to establish such units also in the well recognized medical schools. Medical students were already enrolled in the Medical Enlisted Reserve Corps and were exempted from the draft, but were now ordered to be inducted into active service in the Students' Army Training Corps. The medical schools began at once to carry out the orders and to provide barracks, mess, drill space, etc. The Government was to provide the uniforms, board and tuition of students transferred, and, in addition, to give the students the pay of private soldiers. In only about half of the medical schools, however, had the majority of students been transferred when the armistice was signed and further inductions into the Students' Army Training Corps stopped.

The man-power law extending the requirements of the selective service law was not enacted until August. This made it necessary for the Committee on Education and Special Training to work out the necessarily elaborate plans and carry them into effect in an incredibly short space of time. The medical schools had completed their teaching schedules, and many of them had begun their regular sessions before the program for the transfer of their classes to the Students' Army Training Corps was announced. The deans of the medical schools had, in fact, received no previous intimation regarding the radical nature of the changes finally ordered. The time-consuming routine of changing the students from a civilian to a military status—questionnaires, physical examinations, inductions, waiting in line, etc.—took up most of the students' time which otherwise would have been spent in study. The placing of six hours a week of military training into an already overcrowded schedule added to the confusion, which, in many instances, was further increased by a temporary conflict between the military officer who was supreme and educational officers. For example, drill hours were fixed at the time set for laboratory or clinical courses. Orders through military channels were finally sent to the commanding officers in medical schools where conflicts occurred to make the hours for military drill conform with the teaching schedules. Other difficulties, such as the assignment of students to guard duty, to raking leaves, to kitchen police duties and the like, were also settled by similar orders. Again, the giving up of quiet rooms at their homes or in other private dwellings and the living in barracks, where no provision had been made for study, took away from the students their opportunity for effective work. To cap the climax came the epidemic of influenza, which required the closing of many of the schools. As a consequence, during the time that has elapsed since the opening of the college session, little or no effective teaching has been done in many of the medical schools in which active military training had been established. The difficulty was not due so much to the military training as to the requirement that the students live in barracks and the failure to have the drill hours fit in with the class schedule.

The termination of the war takes away the necessity for further interfering with medical schools, and it is highly important that these institutions be restored to their pre-war status at the earliest possible moment. The orders issued thus far by the War Department indicate a rapid demobilization, and it is probable that in both pre-medical and medical courses the Students' Army Training Corps will soon be discontinued. The medical colleges, therefore, should arrange to extend their sessions for a month or six weeks into next summer so as to make up most if not all of the time that has been lost. If this is not done, the students' ability to secure licenses after they have completed the medical course will be jeopardized. State boards generally have expressed their willingness to make liberal allowances to meet such educational measures as might be necessary in the national emergency. Now that the emergency has disappeared, however, and there is still opportunity to make up the time lost, state boards should not be expected to relax the educational standards provided for in the practice acts. Efficient teaching in the medical schools should be at once restored.—*Journal A. M. A.*, for November 23.

